# Insights into Mechanisms of Chronic Neurodegeneration

**DOI:** 10.3390/ijms17010082

**Published:** 2016-01-12

**Authors:** Abigail B. Diack, James D. Alibhai, Rona Barron, Barry Bradford, Pedro Piccardo, Jean C. Manson

**Affiliations:** 1 The Roslin Institute and R(D)SVS, University of Edinburgh, Easter Bush, Midlothian EH25 9RG, UK; rona.barron@roslin.ed.ac.uk (R.B.); barry.bradford@roslin.ed.ac.uk (B.B.); pedro.piccardo@roslin.ed.ac.uk (P.P.); jean.manson@roslin.ed.ac.uk (J.C.M.); 2 National CJD Research and Surveillance Unit, University of Edinburgh, Western General Hospital, Edinburgh EH8 9JU, UK; james.alibhai@ed.ac.uk

**Keywords:** neurodegeneration, prion, transmissible spongiform encephalopathies (TSE), protein misfolding, proteinopathies

## Abstract

Chronic neurodegenerative diseases such as Alzheimer’s disease (AD), Parkinson’s disease (PD), and prion diseases are characterised by the accumulation of abnormal conformers of a host encoded protein in the central nervous system. The process leading to neurodegeneration is still poorly defined and thus development of early intervention strategies is challenging. Unique amongst these diseases are Transmissible Spongiform Encephalopathies (TSEs) or prion diseases, which have the ability to transmit between individuals. The infectious nature of these diseases has permitted *in vivo* and *in vitro* modelling of the time course of the disease process in a highly reproducible manner, thus early events can be defined. Recent evidence has demonstrated that the cell-to-cell spread of protein aggregates by a “prion-like mechanism” is common among the protein misfolding diseases. Thus, the TSE models may provide insights into disease mechanisms and testable hypotheses for disease intervention, applicable to a number of these chronic neurodegenerative diseases.

## 1. Introduction

Chronic neurodegenerative diseases encompass a number of protein misfolding diseases, which can be both heritable and sporadic. These include Alzheimer’s disease (AD), Parkinson’s disease (PD) and Transmissible Spongiform Encephalopathies (TSEs) or prion diseases. During the course of these diseases, a host-encoded protein misfolds and accumulates in the central nervous system (CNS). Multiple forms of misfolded proteins can be identified in the CNS ranging from small oligomeric structures through to large amyloid deposits [1,2,3,4,5]. The neurotoxicity of the different forms of misfolded proteins has been intensively debated and current literature favours the oligomeric structures as being the more neurotoxic [6,7,8,9,10,11,12,13,14]. The pathogenesis of these diseases is not clear since protein deposits can also be found in apparently healthy individuals [15]. Diagnosis of these diseases often occurs at the latter stages when clinical signs become evident; however, therapeutic interventions have little effect at this stage [16,17,18]. Moreover, the early stages of these diseases are not yet well defined and, thus, few therapeutic targets have been identified in the pre-clinical or early clinical phases of disease.

Traditionally, TSEs have been considered distinct from the other neurodegenerative diseases due to their infectious nature. The nature of the infectious agent has been studied for many decades in numerous large and small animal models, leading to the “prion hypothesis”. This hypothesis predicts that an abnormal conformer of the prion protein is able to bind to the normal conformer, PrP^C^, and induce changes in conformation resulting in an auto-catalytic reaction, which produces abnormal isoforms of the prion protein [19]. Moreover, these abnormal conformers spread both within an individual and between individuals. Originally, these abnormal isoforms (PrP^Sc^) were recognised due to their resistance to proteinase K (PK), but as detection techniques and our understanding of the abnormal protein has increased, so has the range of abnormal isoforms, such as PK-sensitive forms of PrP^Sc^, oligomeric PrP, protofibrils and amyloid fibrils, *etc.* It is still unclear which of these specific conformers may be associated with neurotoxicity and TSE infectivity. 

This “prion-like” spread of protein within the CNS has been more recently observed in a number of non-prion disease associated proteins such as amyloid-beta (Aβ) [20,21], tau [22,23,24,25] and α-synuclein [26,27]. The host protein is considered critical to this cycle of formation and spread. Transmission of a misfolded protein has also been described from one individual to another in a number of experimental models and, more recently, in patients [28,29]. However, to date, there is no epidemiological evidence for inter-individual transmission of diseases associated with proteins other than PrP [30]. Such studies indicate the need to understand the role of the misfolded protein in the infectious and neurotoxic process, in order to accurately assess the risk to individuals and populations from the protein misfolding diseases. This review will describe the progress that has been made using the TSE models in understanding the mechanisms of chronic neurodegeneration and the role of misfolded protein in disease.

## 2. *In Vitro* Modelling of Protein Misfolding

**Figure 1:**
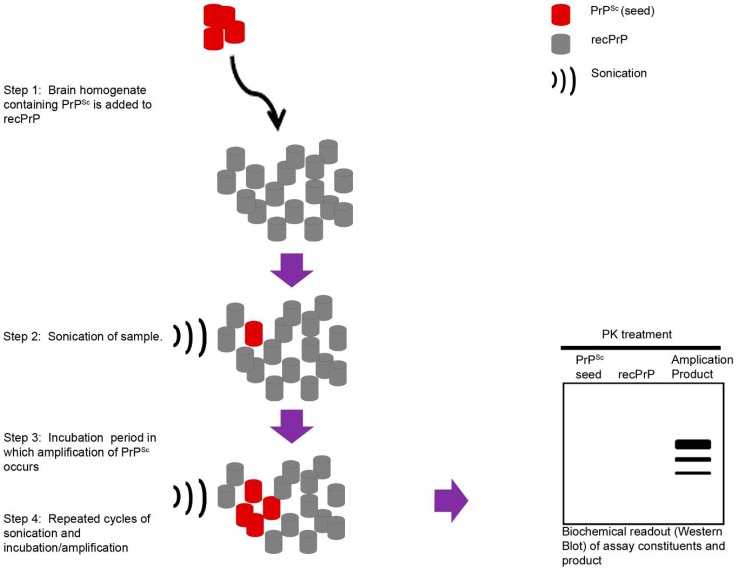
Diagrammatic representation of cell-free conversion assays. A small quantity of a brain homogenate containing PrP^Sc^, usually at quantities lower than is normally detectable when immunoblotting, is added to a pool of “normally” folded host-encoded protein. This can be either purified recombinant proteins (recPrP) or an uninfected brain homogenate. Briefly, the assays undergo periods of incubation, whereby the PrP^Sc^ seeds can interact with recPrP and cause protein misfolding and aggregation. Intermittently, periods of sonication are used to break down larger aggregates to allow further conversion of recPrP to PrP^Sc^. The assay products are then assessed using Western blot analysis. These systems result in a large accumulation of PrP^Sc^, which have occurred in a cell-free system, and, as the result of protein-templated conversion directed from the initial extremely small quantity of PrP^Sc^ added to the reaction in the first instance.

The TSEs have provided us with invaluable *in vitro* systems to assess very small amounts of a misfolded protein in a particular tissue or brain region. These assays have been developed as diagnostic tools but also provide highly sensitive assay systems to probe disease mechanisms. The initial studies demonstrated that enriched preparations of PrP^Sc^ containing brain homogenates, added to recombinant PrP (recPrP), could induce the formation of small quantities of PrP aggregates [31]. Importantly, this reaction occurred in purified recPrP preparations, and, therefore, demonstrated the ability of cell-free protein-templated conversion (Figure 1). The Protein Misfolding Cyclic Amplification (PMCA) assay was developed, which involved cycles of sonication to break down aggregates and incubation to allow further amplification. Additionally, normal uninfected brain homogenate was used to provide PrP^C^ substrate rather than recPrP [32]. Using the PMCA assay, others have identified non-PrP protein factors, which could be important in the conversion mechanism. For example, following the addition of polyanionic compounds (e.g., RNA) to purified PrP^C^, in the absence of misfolded prion protein as a seed, *de novo* generation of PK-resistant PrP (PrP^Res^) was observed [33,34]. Moreover, when experimentally inoculated into mice, this *de novo* generated PrP^Res^ initiated a prion-like disease [35]. A further study then demonstrated high levels of infectivity resulting from the amplification of recPrP in the presence of lipids purified from murine liver [36]. However, evidence from another study using recPrP with physico-chemical characteristics similar to those detected in PrP^Sc^ extracted from infected animals showed no evidence of infectivity [37]. 

It remains unclear whether co-factors are involved in the conversion mechanism *in vivo*. However, understanding that specific types of lipids could play important roles in PrP conversion may be especially relevant when considering the cell-to-cell prion-like spread. As targeting of misfolded proteins, to our current understanding, is highly specific and occurs in a pattern resembling neuronal connectivity, the differences in molecular composition of neurons, specifically at the synapse, between neuroanatomically distinct brain regions, could prove important in defining the mechanism of protein-templated conversion. 

These assay systems have now been extended beyond the measurement of PrP^Sc^ into other misfolded proteins such as α-synuclein and Aβ [38,39]. Roostaee *et al.* [38] showed that a modified PMCA protocol could be used to amplify α-synuclein. The use of PMCA suggests that, as with PrP, interaction between aggregates of pathological α-synuclein and soluble α-synuclein is sufficient to initiate or seed formation of α-synuclein and is thus evidence for self-propagation [38]. Furthermore, PMCA has been adapted for the detection of Aβ oligomers, with initial studies demonstrating that it can distinguish between AD and non-AD patients with a specificity of 90% using cerebrospinal fluid [39]. Current research trends across many neurodegenerative diseases attempt to define spread of misfolded proteins using relatively insensitive detection methods, such as immunohistochemistry or silver staining (reviewed by [40]). In light of the development of these assays to study prion-conversion and the increasing understanding that such assays can be utilised for detection of α-synuclein or Aβ, cell-free conversion assays have the potential to gain further insight into the role of the misfolded proteins in neurodegeneration. 

## *3. In Vivo* TSE Models of Chronic Neurodegeneration

When animals are experimentally infected with a TSE, this represents the “start-point” of the disease process. Well defined time course studies can then be conducted to investigate the disease process from the disease initiation through preclinical and clinical phases of disease [41]. An extensive number of transgenic models have been produced with alterations in the prion protein gene including point and insertional mutations, alterations in the glycosylation status of PrP and alterations in the species from which the PrP is derived. These models range from the gene targeted knock-in models to standard microinjection models overexpressing PrP at various different levels, allowing extensive studies of disease transmission both within and between species [42,43,44,45]. 

PrP^C^ has been demonstrated to be an absolute requirement for disease since, in its absence, mice are resistant to TSEs [46,47] although infection can persist in the PrP knockout (PrP^−/−^) mice for up to 600 days (Manson, personal communication). PrP^−/−^ mice which have wild type (PrP^+/+^) tissue grafts into the brain have demonstrated TSE specific histopathologic alterations of PrP^+/+^ graft tissue but preservation of host PrP^−/−^ neurons when experimentally infected with a TSE [48]. This observation demonstrates that accumulation of PrP^Sc^ is not neurotoxic in the absence of endogenous PrP^C^. Models in which the sequence of the host PrP gene has been altered have also demonstrated the importance of host sequence in determining the incubation time and pathological lesions in the disease process [49,50,51,52,53,54,55]. Moreover, the glycosylation status of host PrP has been shown to have a remarkable influence on susceptibility and resistance of the host to disease [56,57,58,59].

## 4. Time Course Studies of TSE 

Understanding the pathophysiological progression of chronic neurodegenerative diseases is of importance in determining how an abnormally folded protein may cause disease. Although incubation periods and the timing of events may vary between TSE models, the models themselves recapitulate pathologies observed in natural diseases such as spongiform degeneration of the brain, neuronal loss, abnormal protein deposition, synaptic degeneration and gliosis (reviewed in [60,61]). Behavioural deficits can be observed throughout the incubation period and correlated to pathogenesis of the CNS, and, eventually, overt clinical symptoms of disease are observed. 

**Figure 2:**
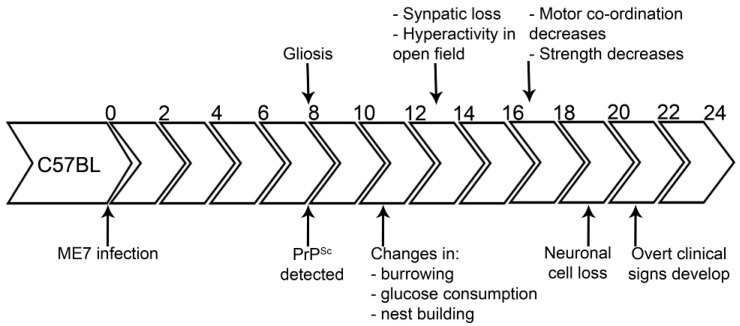
Time course of a TSE infection; ME7/C57BL. The murine TSE strain, ME7 is intracerebrally inoculated into C57BL mice. The total incubation period in this model is approximately 24 weeks. PrP^Sc^ and gliosis can be detected from eight weeks post inoculation. This is followed by changes in behavioural signs (from 10 weeks), synaptic loss (from 12 weeks), neuronal loss (from 19 weeks) and overt clinical signs can be observed from approximately 20 weeks. Data in this figure was obtained from [62,63,67].

Following inoculation with a TSE agent, small accumulations of PrP^Sc^ can be initially detected in restricted regions of the brain, shortly followed or concurrently with glial cell responses (microglia and/or astrocytes), often within the same brain regions. This is followed by synaptic protein loss and electrophysiological deficits. It is around this time that early behavioural deficits may be observed *i.e.*, changes in burrowing behaviour and glucose consumption [62,63,64,65,66]. Indeed, the behavioural deficits can be correlated to synaptic loss in specific brain regions such as the hippocampus and hypothalamus [67,68]. Throughout the time course of disease, behavioural changes become more marked and varied from their first manifestation. Finally, neuronal loss and the clinical onset of disease can be observed. An example of a time course of TSE disease is summarised in Figure 2.

At clinical stages, PrP^Sc^ is usually detected in brain regions undergoing neurodegeneration. The accumulation and aggregation of PrP^Sc^ occurs long before detectable neurodegeneration [63,69] and the spread of misfolded protein between distinct brain regions is thought to determine the specific brain regions that undergo neurodegeneration (reviewed in [40,70]). These studies have led many to conclude that the misfolding of the host protein is the initiating and causative factor of neurodegeneration [71,72,73]. However, a number of findings question the direct relationship between protein misfolding and neurodegeneration. For example, in some TSEs, misfolded PrP accumulates in the brain in some situations unaccompanied by the other typical neuropathological changes or any clinical signs of disease [5,74,75]. Protein accumulation in the brain in the absence of clinical disease has also been observed in AD with accumulation of Aβ-amyloid plaques in the brains of cognitively normal, aged individuals [15]. Thus, the relationship between misfolded proteins and neurodegeneration is not fully established. 

## 5. Neurodegeneration and Protein Misfolding

The accumulation of PrP^Sc^ aggregates is thought to be associated with either a toxic gain of function or loss of normal function [76]. The prevailing hypothesis is that the protein aggregates or seeds trigger a cascade of events leading to neurodegeneration. It has been proposed that the formation of an initial protein assembly, referred to as a nucleation event or seed, catalyzes the conversion of normal protein into a pathologic state. Once seeded, growth of amyloid-like structures is typically exponential, resulting in the formation of macromolecular structures that appear as intra or extracellular deposits used to pathologically define the disease [8,77]. Whether this seeding and spread of the misfolded protein results in a clinical disease, however, may also depend on the host response to the misfolded protein. Recent studies have shown the formation of PrP-amyloid in the absence of other prion-associated pathological changes or clinical signs, indicating that seeded polymerization of misfolded protein aggregates does not always results in neurodegeneration [5,74,75]. Moreover, recent studies by Alibhai *et al.* [78] have challenged the concept that the accumulation and spread of misfolded protein determines neurodegeneration by showing that proteins seeds alone are not pathogenic. This raises the question of what, in addition to misfolded protein, is required for a neurodegenerative cascade, which will be further addressed in Section 6. Glial Cells and Neurodegeneration.

In studies in which transgenic mice overexpressing human forms of amyloid precursor protein (APP) were inoculated with brain homogenates containing human Aβ aggregate, further formation of Aβ aggregates was evident [20]. Other disease-associated protein aggregates, such as tau aggregates, can also experimentally induce and propagate after inoculation into transgenic mice expressing human tau [22,23,24]. Similarly to TSE diseases, the inoculation of α-synuclein containing brain extracts or aggregates into transgenic and wild type mice resulted in clinical disease and α-synuclein deposition [79,80] or clinical disease, cell-to-cell transmission of pathologic α-synuclein and Parkinson’s-like Lewy pathology in anatomically interconnected areas respectively [26]. In addition, aggregation and propagation of endogenous tau were observed in wild type mice inoculated with tau oligomers purified from the brain of an AD patient [25]. Seeding of β-amyloidosis has also been reported in non-human primates injected intracerebrally with human or marmoset brain homogenate containing β-amyloid [81].

Similar to evidence presented in TSEs, this spread of the misfolded protein appears to occur in a pattern resembling neuronal connectivity [22,23,24,25,26,82,83]. Furthermore, peripheral administration of disease-associated aggregates into mice also has the capability to trigger subsequent protein aggregation in the brain [84,85]. The relevance of this recent array of data has led to the interpretation that disease-associated misfolded proteins, such as Aβ, tau and α-synuclein, amongst others, can transmit between cells in a prion-like mechanism (reviewed in [86,87,88]). It is therefore important to understand the implications for these protein accumulations in terms of clinical disease and transmission of disease to another host.

## 6. Glial Cells and Neurodegeneration

The high levels of PrP expression in the neuronal cells of the CNS have led researchers to focus on neuronal cells in defining mechanisms of neurodegeneration in the TSEs. PrP mRNA is found throughout neuronal cells of the brain but with variable levels in different neuronal populations [89,90]. Evidence for a cell autonomous neurodegenerative mechanism has been provided from *in vivo* studies with transgenic mice designed to express PrP^C^ in neurons only, which were shown to be susceptible to TSEs [91]. Conversely, using a model in which PrP^C^ expression was removed from neurons at a specific time point during the course of disease, the disease process appeared to be blocked [92]. The removal of neuronal PrP resulted in the reversal of TSE associated spongiform degeneration of the brain and behavioural deficits in this model [93,94]. Other studies have demonstrated that reduction in neuronal PrP at any point throughout the preclinical phase, right up to clinical onset, resulted in dramatic lengthening of the pre-symptomatic period of the disease, although, in this case, all animals ultimately developed a clinical neurodegenerative disease [95]. This extension of the pre-symptomatic phase in this study, however, indicates that there is a wide therapeutic window prior to the onset of clinical symptoms for possible intervention into these devastating diseases.

The expression of *Prnp* mRNA and PrP protein has also been described in non-neuronal cell types in the CNS [96,97,98], and the accumulation of misfolded protein occurs in non-neuronal cell types within the brain during TSE infection. Transgenic mice expressing PrP only in astrocytes succumb to TSE infection, demonstrating that the expression of PrP^C^ in astrocytes is sufficient to elicit neurodegeneration and a clinical TSE disease [99]. Indeed, the astrocytic production of PrP may play a role in sustaining the disease process in non-transgenic animals.

During the earliest stages of TSE pathogenesis within the CNS, a strong reactive glial response is observed. Astrocytes and microglia have been implicitly linked with the disease process through extensive pathological [66,100] and gene expression analysis [101]. Astrocytes are sensitive to changes in homeostasis, injury to the CNS and the presence of misfolded proteins [102], and undergo a complex response, morphologically characterised by hypertrophy of the cell body and shortening/thickening of processes [103,104]. During TSE, it has been reported that PrP^Sc^ accumulates in astrocytes [105], and, therefore, it could be argued that a loss of astrocyte function could be an important component of the disease process. Astrogliosis is observed prior to functional synaptic deficits and concurrently with the appearance of detectable PrP^Sc^ [63] suggesting astrocyte activation is occurring as a result of misfolded protein. Alternatively, astrocyte activation may occur directly in response to degenerating neurons or other CNS insults [106]. 

An activated morphological response by microglia is also observed early following neuroinvasion or during the early stages after intracerebral inoculation. This morphological response is accompanied by a mixed cytokine response that includes mediators of both potent inflammatory responses e.g., IL-1B, IL6 and anti-inflammatory responses such as TGFB1 and IL10 [107,108]. Inhibition of microglial proliferation results in increases in the TSE incubation period [109] suggesting a potential for therapeutic intervention by modulating the microglial response to disease. 

Genetic knockout (KO) models of genes involved in the innate immune response have been shown to influence the incubation period of TSEs [110]. For example, KO of *Il1r1*, *Ccl2*, *Cxcr3* and *Cd14* result in a prolonged incubation period [111,112,113,114]. KO or knock-down of *Tgfb1*, *IL4*, *IL10*, *IL13* or *Ccr1*, on the other hand, results in a shortened incubation period [111,115,116]. This data demonstrates that altering the innate immune response in specific ways can alter pathogenesis and progression of disease, highlighting an important role of glial cells in the disease process. However, exactly how non-neuronal cells support the disease mechanism is not yet clear and whether their activation occurs in response to degenerating neurons, protein deposition or other CNS insults is still a matter for debate. Understanding the interactions between neuronal and glial cells in the disease process may prove important in defining the mechanisms underlying neurodegeneration 

## 7. Protein Misfolding and Infection 

The exact nature of the infectious entity in TSE has not been determined. Estimates on the size of the most infectious PrP particle have varied [117,118,119,120,121,122,123,124] and co-factors have been suggested that are required for the infectious process [36,125,126,127,128,129,130,131,132,133]. Moreover, while the TSE in general are considered to be infectious, there is a wide range of infectious potential amongst these diseases ranging from the contagious diseases such as Chronic Wasting Disease (CWD) and scrapie [134,135] through to those with zoonotic potential such as Bovine Spongiform Encephalopathy (BSE) [136,137,138] and others which are extremely hard to transmit such as the human forms of disease [139,140,141,142]. Results from the National Institutes of Health series of 300 experimentally transmitted cases into non-human primates show highest transmission rates for iatrogenic and sporadic cases and lower for familial forms of disease [139]. 

There are many strains of TSE agents described by their differences in incubation period, pathology and biochemical properties [143]. The exact definition of these strains has yet to be made, but explanations from nucleic acid [144,145] to the conformation of PrP^Sc^ [146,147,148,149], or host-specific factors other than PrP^C^ have been put forward [150]. The idea for conformation of misfolded protein defining strains has been extended to other non-prion misfolded proteins such as Aβ [151], tau [152] and α-synuclein [153,154] and may go some way to defining different clinical phenotypes that are observed in the associated diseases. 

It has been proposed that sequence identity between PrP^C^ in the host and PrP^Sc^ in the inoculum is central for the efficient transmission of disease between individuals [155]. Others have suggested conformation rather than sequence determines transmission characteristics [149,156]. However, this is not always the case [50,157,158]. Glycosylation of host PrP has been considered to be an important factor [58,59]. Transgenic mice expressing fully or glycosylation deficient PrP isoforms have been exposed to TSE strains with distinct glycosylation patterns, and it has been shown that transmission of TSE agents across species can be profoundly influenced by the glycosylation status of both PrP^C^ and PrP^Sc^ [56,57,159,160].

Transgenic models were further developed to assess the influence of disease-associated mutations in the *PRNP* gene on the development of disease. “Knock-in” transgenic mice with a targeted proline (P) to leucine (L) mutation in the *Prnp* gene (101LL) were generated to model a familial TSE, Gerstmann-Sträussler-Scheinker (GSS) P102L disease [51]. The 101LL mice do not develop any spontaneous TSE but proved to be highly susceptible to several TSE strains. When 101LL mice were inoculated with brain extracts from patients with “typical” GSS P102L (with widespread spongiform degeneration, diffuse and amyloid PrP deposits), they developed neurological signs and replicated high titers of infectivity in the brain. However, unexpectedly, no PrP^Sc^ was observed in the brain of these mice by Western blot analysis [161]. Similar cases of TSE disease in the absence of detectable PrP^Sc^ was reported by Lasmezas *et al.* with the transmission of BSE to mice [162] and in transmission of atypical/Nor98 scrapie from peripheral tissues with no detectable PrP^Sc^ [163]. Recent studies using highly sensitive detection systems (such as PMCA) for the identification of small amounts of PrP^Sc^ showed prion infectivity in peripheral tissues of terminally diseased BSE field cases in the absence of detectable PrP^Sc^ [164]. The importance of these studies has been to question the relationship between PrP^Sc^ and infectivity, and, indeed, these studies lead to the suggestion that both PK resistant and PK sensitive forms of the prion protein had infectious potential [165,166,167].

**Figure 3:**
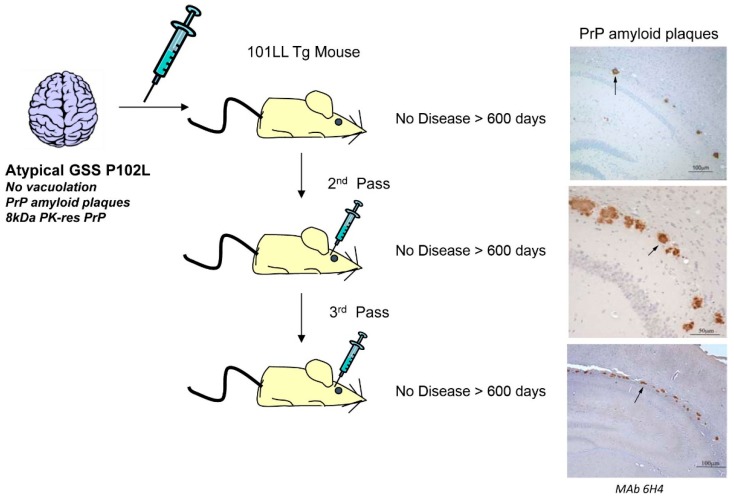
Transmission of atypical GSS P102L into 101LL mice. Intracerebral inoculation of partially purified PrP^Sc^ (brain homogenate) from a patient with atypical GSS P102L into 101LL transgenic mice resulted in multicentric PrP-positive plaques in the corpus callosum and periventricular zone of 101LL mice at >600 days post inoculation (dpi). Two subsequent subpassages using infected 101LL mouse brain homogenate also showed multicentric PrP-positive plaques, again at 600 dpi. No clinical signs of prion disease or vacuolar pathology were observed in any mice despite the presence of PrP-amyloid plaques. PrP-amyloid stained with anti PrP Mab 6H4 [169]. Arrows indicate PrP amyloid plaques.

The relationship between misfolded PrP and infection has been further confounded by the inoculation of partially purified PrP^Sc^ from a patient with atypical GSS P102L into 101LL mice, which resulted in the accumulation of large multicentric amyloid plaques in restricted areas of the brain but no clinical disease or spongiform degeneration of the brain. Subsequent sub-passages of the brain material from these mice into 101LL mice showed the presence of PrP amyloid without any clinical signs of disease (Figure 3) [5]. These studies demonstrate that infectivity may not always be directly related to PrP pathogenicity, and, moreover, protein accumulation in the CNS does not always lead to a clinical disease [5,74,168]. This raises the question of which PrP^Sc^ isoform(s) are neurotoxic, which are associated with infectivity and what is the molecular distinction between these two PrP^Sc^ species.

As discussed in Section 5. Neurodegeneration and Protein Misfolding, numerous studies have provided evidence that non-prion misfolded protein can induce aggregate formation following intra-cerebral inoculation into mice and non-human primates [20,22,23,24,25,26,27,81]. The misfolded proteins are proposed to transmit between cells in a prion-like mechanism; this also infers that they may have the potential to be infectious *i.e.*, transmit between individuals. Murine transmission studies involving peripheral inoculations of misfolded protein have provided evidence that peripheral challenge with Aβ and tau can cause protein aggregation in the brain [84,85]. These studies used overexpression mouse models, which have the potential to accelerate disease progression, develop spontaneous disease and the expression of protein can vary throughout the body, making interpretation of the studies difficult. Jaunmuktane *et al.* observed the presence of Aβ in individuals who had received human pituitary-derived growth hormone prompting consideration that iatrogenic routes of transmission may be relevant to Aβ and other non-prion misfolded proteins as well as TSEs [28]. However, there is no epidemiological evidence to support this having occurred [30]. Understanding the association between a misfolded protein and neurotoxicity or infectivity will aid us in determining whether all protein misfolding diseases represent a risk to public health.

## 8. Conclusions

In view of the central role of the host-encoded proteins such as PrP in TSEs, amyloid precursor protein (APP) in AD and microtubule associated protein (tau) in AD, PD and tauopathies, these neurodegenerative diseases are grouped together as protein misfolding diseases [20,21,29,170,171,172,173,174,175,176,177,178]. Increasing numbers of publications describe the spread of AD, PD and other diseases by what is called a “prion-like” mechanism. The data focus on the cell-to-cell spread of misfolded protein aggregates as a general pathogenic phenomenon. Most neurodegenerative disorders (e.g., AD, PD) have previously not been considered to be infectious diseases. Indeed, there is no epidemiological data to suggest that they are infectious [30]. It is important, therefore, to understand when a misfolded protein has the potential to lead to clinical disease and to be transmitted between individuals via natural routes of transmission, which should be considered a different scenario from cell-to-cell spread within an individual. Thus, determining the difference between protein aggregates associated with neurotoxicity and infectivity and those aggregates that are benign is essential to determine when diseases associated with protein misfolding are threats to individuals and to public health. The TSE systems have given some insights and invaluable tools to study these disease mechanisms; in particular, early events in disease pathogenesis can be elucidated. It has been shown that both agent and host define the infectious nature of these diseases. Moreover, glial and neuronal cells are important to the disease process. While these studies are pointing to new avenues for therapeutic intervention, we are still some way from providing a clear understanding of these complex disease processes. However, by using a concerted approach to the family of prion-like diseases rather than considering each disease in isolation, we might provide more rapid progress to understanding the underlying mechanisms of the disease process and develop novel intervention strategies applicable to all forms of protein misfolding diseases.
